# Long‐term quality of life & functional outcomes after treatment of oropharyngeal cancer

**DOI:** 10.1002/cam4.3599

**Published:** 2020-12-04

**Authors:** Susanne I. Scott, Anne Kathrine Ø. Madsen, Niclas Rubek, Birgitte W. Charabi, Irene Wessel, Sara Fredslund Hadjú, Claus V. Jensen, Sarah Stephen, Joanne M. Patterson, Jeppe Friborg, Kathrine A. Hutcheson, Henrik Kehlet, Christian von Buchwald

**Affiliations:** ^1^ Department of Otorhinolaryngology, Head and Neck Surgery & Audiology Rigshospitalet Copenhagen University Hospital Copenhagen Denmark; ^2^ Department of Occupational Therapy and Physiotherapy, Rigshospitalet Copenhagen University Hospital Copenhagen Denmark; ^3^ Cancer Late Effects Research Unit (CASTLE) Department of Oncology Rigshospitalet Copenhagen Denmark; ^4^ Department of Diagnostic Radiology Rigshospitalet Copenhagen University Hospital Copenhagen Denmark; ^5^ Department of Speech, Voice and Swallow ENT Freeman Hospital Newcastle upon Tyne UK; ^6^ School of Health Sciences / Head and Neck Cancer Centre University of Liverpool Liverpool UK; ^7^ Department of Oncology, Rigshospitalet Copenhagen University Hospital Copenhagen Denmark; ^8^ Department of Head and Neck Surgery Division of Radiation Oncology The University of Texas MD Anderson Cancer Center Houston TX USA; ^9^ Section for Surgical Pathophysiology Rigshospitalet Copenhagen University Hospital Copenhagen Denmark

**Keywords:** dysphagia, functional outcomes, oropharyngeal cancer, quality of life, radiotherapy, saliva, shoulder function, transoral robotic surgery

## Abstract

Functional and QoL outcomes were compared longitudinally in a cohort of patients treated for oropharyngeal squamous cell carcinoma (OPSCC) with primary transoral robotic surgery (TORS) or radiotherapy (RT). Forty‐four patients undergoing primary TORS (n = 31) or RT (n = 13) for any stage OPSCC were included. Only low‐stage disease was treated with TORS. Functional outcomes were: salivary flow rate, image‐based swallowing function, and a self‐reported 10‐point scale comparing current swallowing function to baseline (CvB scale). QoL was assessed with European Organization for Research and Treatment of Cancer Quality of Life Questionnaire Core (EORTC QLQ‐C30), Head & Neck Module (EORTC QLQ‐HN35), and MD Anderson Dysphagia Inventory (MDADI). Shoulder impairment was assessed with Neck Dissection Impairment Index (NDII) and Oxford Shoulder Score (OSS). In the RT group, salivary flow rates had significantly declined at 12‐month follow‐up, with the biggest declines in QoL subscale scores recorded in the RT group for dry mouth and sticky saliva. Swallowing function on imaging studies was overall good, with no severe dysphagia within 1 year although, both treatment groups showed significant deterioration relative to baseline at the 12‐month follow‐up with increased DIGEST scores and pharyngeal retention. Shoulder impairment was rare at 1 year in both groups. A comprehensive examination of this cohort treated for OPSCC showed overall good functional and QoL outcomes 1 year after treatment. However, persistent impairment was seen in both groups with regards to swallowing function. In the TORS group, at 12‐months follow‐up, the QoL questionnaires showed worse scores in only one subscale (sticky saliva).

## Graphical abstract

In this detailed study of functional and quality of life, 1 year after treatment of oropharyngeal squamous cell carcinoma with either transoral robotic surgery (n = 31) or radiotherapy (n = 13). Functional outcomes were overall good, but persistent impairment was seen in both groups particularly with regards to swallowing function. Worsening in salivary outcomes were seen after treatment with radiotherapy.

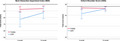

## 1 INTRODUCTION

The incidence of oropharyngeal cancers, including oropharyngeal squamous cell carcinomas (OPSCC), is increasing mainly due to a sharp increase in HPV positive cancers in many countries.^1^, ^2^ With a majority of OPSCC being HPV positive and, as HPV‐positive OPSCC has a better prognosis, more patients become long‐term cancer survivors, living with chronic adverse treatment side‐effects.

With similar survival rates between primary surgical and primary oncological treatments,^2^, ^3^, ^4^ there is a need for comprehensive high‐quality trials investigating the complications and adverse effects of available treatment options, particularly with regards to long‐term functional outcomes.

Considering this, we report a single‐institutional observational prospective cohort study investigating functional and quality of life (QoL) outcomes in the first 12 months after treatment with either primary transoral robotic surgery (TORS group) or primary radiotherapy (RT group). This study was designed as a descriptive hypothesis‐generating feasibility study, and therefore, cannot be used to compare treatment modalities. We present data for each group to investigate differences in modality specific adverse effects.

## 2 METHODS

Patients diagnosed with OPSCC, regardless of stage, were prospectively enrolled from May 2017 to October 2018 at the Department of Otorhinolaryngology, Audiology and Head & Neck Surgery at Copenhagen University Hospital (Rigshospitalet), Denmark.

Patients with verified OPSCC, WHO performance stage 0–2, in the absence of distant metastasis, previous head and neck cancer or radiotherapy to the head and neck were eligible to participate.

TORS is considered an experimental treatment for OPSCC in Denmark and all patients in this cohort were offered primary oncological treatment. Patients with low‐stage disease (T1‐T2, N0‐N1, M0 according to the union for international cancer control, IUCC 7th, edition) who were assessed as operable without trismus or radiological signs of extra capsular extension in metastatic lymph nodes were also offered TORS and ipsi‐ or bilateral selective neck dissection. All except one patient, who were offered TORS, chose this treatment, which means that included patients treated with primary RT, were patients who did not qualify for TORS.

The study was conducted in accordance with the Declaration of Helsinki and was approved by the local ethical committee (H‐1‐2014‐033 and H‐17015164). Written informed consent was obtained. The study protocol has been published on clinicalTrials.gov (NCT03418909).

### 2.1 Treatment

#### 2.1.1 Surgery

The da Vinci SI Surgical System (Intuitive Surgical Inc) was used for all TORS procedures. Resection margins of >2 mm or supplementary free margins were considered negative margins.^5^, ^6^ TORS was performed with concurrent neck dissection––if not already performed as part of our “cancer of unknown primary” diagnostic algorithm. For tonsillar malignancies, a unilateral selective neck dissection was performed. A bilateral neck dissection was performed for midline malignancies; base of tongue and soft palate. Selective neck dissection was performed according to local guidelines. Adjuvant RT was recommended in case of positive margins, extra nodal extension (ENE), or more than one involved lymph node.

#### 2.1.2 Radiotherapy

In this study, patients with biopsy confirmed T‐stage >2 and/or N‐stage >1 with M‐stage =0 (according to UICC7) were offered RT (with or without chemotherapy). RT was delivered using volumetric arc therapy (VMAT). Target volumes consisted of a gross tumor volume (GTV) with tumor tissue verified by imaging data and clinical examination, including macroscopically involved lymph nodes. This was encompassed by a clinical target volume, CTV1, with a 5–10 mm margin from GTV. If a 5 mm expansion was used for CTV1, a CTV2 encompassed CTV1 with a concentric margin of 5 mm was added. CTV3 encompassed CTV2 and also included low risk regional elective lymph node regions. Accelerated radiotherapy with 6 fractions/week was standard. CTV1 received 68 Gy/34 fractions if the macroscopic tumor exceeded 4 cm in largest diameter, otherwise 66 Gy/33 fractions. A minimum of 60 Gy was prescribed to CTV2, and 50 Gy to CTV3. Fit patients with T3‐T4 or N+ disease were offered concurrent chemotherapy with Cisplatin 40 mg/m^2^ weekly. Nimorazole, a radiosensitizer, was offered all patients receiving primary radiotherapy.

Adjuvant RT comprised 66 Gy to areas of residual tumor, involved resection margin or ENE, 60 Gy to the remaining tumor‐bed and 50 Gy to the elective volumes as described in primary radiotherapy. Fit patients with positive margins (<2 mm) or ENE were offered concurrent chemotherapy with Cisplatin 40 mg/m^2^ weekly.

### 2.2 Data collection

Pretreatment data included age, sex, smoking history, American Society of Anesthesiologists (ASA) score, and weight. After treatment start, the following data were collected: uni‐ or bilateral neck treatment, aspiration pneumonias, tracheotomies, hemorrhages, re‐resections, adjuvant treatment, admissions/re‐admissions including indication for admission, recurrences, progression, and death.

Study parameters, including QoL outcomes, swallowing function, and salivary flow rates, were assessed pretreatment (baseline) as well as twice posttreatment. Shoulder function was not assessed at baseline. The first posttreatment follow‐up was performed around 3 months (3‐month follow‐up) after *last* treatment day (mean 99 days, range 55–195) including adjuvant treatment if received. The second posttreatment follow‐up was performed 12 months (12‐month follow‐up) after treatment *start* (mean 377 days, range 335–447). Last treatment day was chosen as the anchoring point for the first follow‐up as RT takes time to administer and adverse effects accumulate over the treatment period and 3 months after treatment start would be 1½–2 months after completion of treatment for patients receiving RT. The anchoring point was treatment start for the second follow‐up to align with survival and recurrence data.

### 2.3 Functional outcome measures

#### 2.3.1 Salivary flow rate

Salivary flow rate was assessed using the drooling method^7^ (see Appendix for details). Flow rates were calculated in ml/min.

#### 2.3.2 Imaging based swallowing assessments

Patients were examined using fiberoptic evaluation of swallowing (FEES) and video fluoroscopy (VF) on the same day. The same boluses were administered at both examinations: 15 ml water (International Dysphagia Diet Standardisation Initiative, IDDSI, level 0), 15 ml of moderately thickened water (IDDSI level 3) and one bite of white bread (IDDSI level 7).^8^

VF was evaluated using DIGEST. DIGEST grades are based on patterns of penetration/aspiration and pharyngeal residue to derive an overall severity grade of dysphagia that aligns to CTCAE toxicity grading framework; from grade 0 (best function), grade 1 (mild), grade 2 (moderate), grade 3 (severe), to grade 4 (profound/life threatening dysphagia, worst function).^9^

FEES was analyzed using the Yale Pharyngeal Residue Severity Rating Scale (Yale) for residue at the vallecula and the piriform sinus,^10^ and was assessed after the last swallow of the bolus.

#### 2.3.3 CvB scale

As a crude guide to subjective swallowing function patients were asked at 3‐ and 12‐month follow‐up: “If your pre‐illness (precancer) swallowing function was 10 out of 10. What is it now on a scale from 1 to 10, where 1 is the worst imaginable function?”. By predefining 10 out of 10 as the pre‐illness function, the scale only measures patients’ perceived deterioration in swallowing function. The scale, named after its inventor (and co‐author), was invented for this study, and has not previously been used or validated.

#### 2.3.4 MD Anderson Dysphagia Inventory (MDADI)

MDADI is a 20‐question swallowing related QoL questionnaire. It can be summarized in two overall functional scores: one based on a single question (global score) and the other based on the remaining 19 questions (composite score). The composite score ranges from 20 to 100 with a higher score representing better QoL.^11^ A 10‐point change in score is generally considered a clinically relevant difference.^12^ The 19 questions making up the composite score can also be reported in three subscales (emotional, functional and physical). As part of the Swedish translation of the MDADI four additional questions were added; labeled X1‐X4. These were included in the validated Danish translation and the results are displayed as a subscale termed X‐scale (see Appendix for full questions).^13^

#### 2.3.5 EORTC‐C30 and EORTC‐H&N35

EORTC‐C30 and H&N35 comprises several scales that can be divided into functional, global health, and symptom scales. All scales and single‐item measures are normalized to range from 0 to 100, where a high score represents high QoL for the functional scales (physical, emotional, cognitive, and social) and global health status. Conversely, a high symptom scale score indicates a high level of symptomatology (low QoL). The minimal clinically important difference was set as 10% of the maximum instrument score (i.e. 10 points).^14^

#### 2.3.6 Neck Dissection Impairment Index (NDII)

An unvalidated Danish translation of the neck dissection impairment index, version 1 was used.

NDII comprises 10 questions all of which are rated on a 5‐point Likert scale. The scores can be summarized in a total score ranging from 0 to 100, where a high value represents good function.^15^ An 18‐point change was considered clinically meaningful.^16^

#### 2.3.7 Oxford shoulder score (OSS)

Similar to the NDII patients were asked to complete the OSS at 3‐ and 12‐month follow‐up.

The OSS was developed to assess all types of shoulder conditions (apart from instability). It comprises 12 questions scored from 0 (poor) to 4 (best). The 12 question scores are added to a total score that ranges from 0 to 48, with a high score representing good function.^17^

### 2.4 Statistical analysis

Statistical analyses were conducted using SPSS, version 25 (IBM Inc). All outcomes were compared for each treatment group between baseline and 12‐month follow‐up, except for shoulder scores. When differences in scores were not normally distributed (DIGEST and FEES related scores: PAS and Yale) nonparametric signed rank test was used with the effect size (r) and 95% CIs. The magnitude of the effect size was interpreted in accordance with Cohen Benchmarks with *r* = 0.10, small; *r* = 0.30, medium; and *r* = 0.50, large.^18^ For outcomes where the differences were normally distributed (salivary flow rates, CvB, MDADI, EORTC‐C30 and ‐H&N35 scores) comparison was made using the dependent *t* test.

Missing data in QoL questionnaires and functional swallowing assessments were not statistically replaced. However, for OSS only, and in accordance with the developer's guidelines one or two missing values were replaced with an average of the remaining question scores.^17^

## 3 RESULTS

In the study period 110 patients met the inclusion criteria, 32 patients undergoing TORS were included versus 13 patients undergoing RT. Of the remaining 65 patients 31 received RT in a different hospital, and 34 received RT at our hospital but declined to participate. Informed consent was needed for inclusion.

Patient characteristics are reported in Table 1. The most common primary tumor site was palatine tonsil (68%) followed by base of tongue (25%) and soft palate (7%).

**TABLE 1:** Patient characteristics

	All n = 44	TORS n = 31	RT n = 13
Age median (range)	58 (46–78)	59 (46–78)	58 (51–72)
Sex			
Male	33 (75%)	22 (71%)	11 (85%)
Female	11 (15%)	9 (29%)	2 (15%)
Primary tumor location			
Palatine tonsil	30 (68%)	22 (71%)	8 (62%)
Base of tongue	11 (25%)	6 (19%)	5 (38%)
Soft palate	3 (7%)	3 (10%)	0
Clinical TNM			
T1 n (%)	21 (48%)	15 (48%)	6 (46%)
T2 n (%)	20 (45%)	16 (52%)	4 (31%)
T3 n (%)	1 (2%)	0	1 (8%)
T4 n (%)	2 (5%)	0	2 (15%)
N0 n (%)	16 (36%)	16 (52%)	0
N1 n (%)	27 (61%)	15 (48%)	12 (92%)
N2 n (%)	1 (2%)	0	1 (8%)
UICC8 stage, n (%)			
I	41 (93%)	31 (100%)	10 (77%)
II	1 (2%)	0	1 (8%)
III	2 (5%)	0	2 (15%)
HPV positive Missing RT = 1	36 (82%)	24 (77%)	12 (92.3%)
p16 positive Missing RT = 1	36 (82%)	24 (77%)	12 (92.3%)
Smoking status			
Never smoker	14 (32%)	11 (35%)	3 (23%)
0–10 pack years	5 (11%)	3 (10%)	2 (15%)
>10 pack years	25 (57%)	17 (55%)	8 (62%)
Radiotherapy	18 (41%)	5 (16%)	13 (100%)
Chemotherapy			
Cisplatin	13 (30%)	4 (13%)	9 (69%)
Carboplatin		1 (3%)	0
Nimorazol	15 (34%)	2 (7%)	13 (100%)
ASA			
ASA 1	21 (48%)	13 (42%)	8 (62%)
ASA 2	18 (41%)	16 (52%)	2 (15%)
ASA 3	5(11%)	2 (6%)	3 (23%)
Deaths			
Disease specific	1		1
Unrelated	1	1	

Abbreviations: ASA, American Society of Anesthesiologist Classification; cTNM, clinical TNM stage (UICC 7); HPV, Human Papilloma Virus; RT, radiotherapy; TORS, Trans Oral Robotic Surgery.

In the TORS group, five (16.1%) had hemorrhages requiring treatment (range perioperatively—17 days), one of which was an arterial bleed upon extubation, an emergency tracheotomy was performed to regain control of the airway and reverted when hemostasis was achieved. Fourteen (45%) had midline malignancies (base of tongue or soft palate) and received bilateral neck dissection. Four (12.9%) were treated for aspiration pneumonia (range 5–12 days postoperatively). One patient (3.2%) was upstaged during TORS to a T4 and referred to adjuvant chemoradiotherapy (CRT). Four (12.9%) were found to have ENE on final pathology and were referred to adjuvant CRT. One declined and presented with N‐site failure at 6 months at which time he received salvage CRT. The average length of stay after TORS was seven days (range 4–46 days).

In the RT group, 13(100%) completed radiotherapy (66–68 Gy), nine (69%) tolerated Cisplatin, and all 13 received Nimorazole. Nine (69.2%) had bilateral neck radiation, and three (23.1%) had selective neck dissections for suspected recurrence within 12‐month follow‐up (range 2–9 months). Seven (53.8%) were admitted to hospital with overnight stays a total of 12 times, 10 admissions were due to side‐effects of treatment (nausea, vomiting, pain, dehydration, and reduced oral intake), one due to neutropenic fever and one as part of investigations for possible recurrence. Four (30.1%) did not complete the 3‐month follow‐up, one of whom declined follow‐up after completing treatment.

The mean follow‐up time was 26 months (range 17–34). Two patients died within the follow‐up period.

### 3.1 Salivary flow rate

**FIGURE 1:**
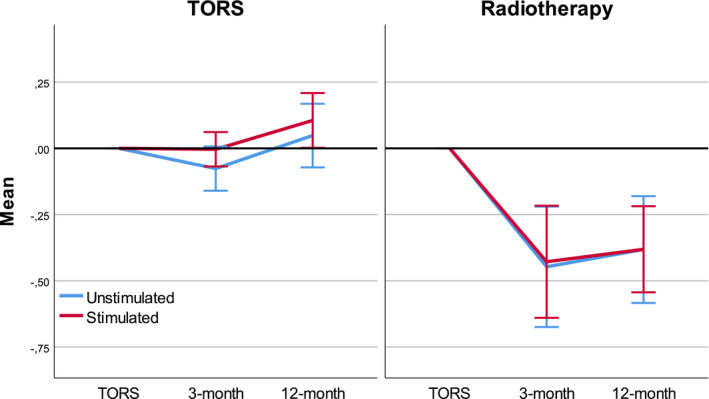
Change in mean salivary flow from follow‐up to baseline. Negative values represent a mean decline in salivary flow rate (ml/min)

Changes in mean salivary flow rate compared to baseline (where a low value represents a decrease in flow rate) are shown in Figure 1.

When comparing baseline to 12‐month follow‐up, the TORS group had a significant increase in stimulated flow rate, with a mean change of 0.1 ml/min (95% CI of change 0.0–0.2, *p* = 0.046). In the RT group there was a significant decline in both stimulated and unstimulated flow rates. With a mean decline of 0.38 ml/min in both flow rates, the 95% CI of the change was −0.5 to −0.2 in the stimulated and −0.6 to −0.2 in the unstimulated flow rate with *p* values of >0.001 and 0.002, respectively.

### 3.2 Imaging assessed swallowing function

**FIGURE 2:**
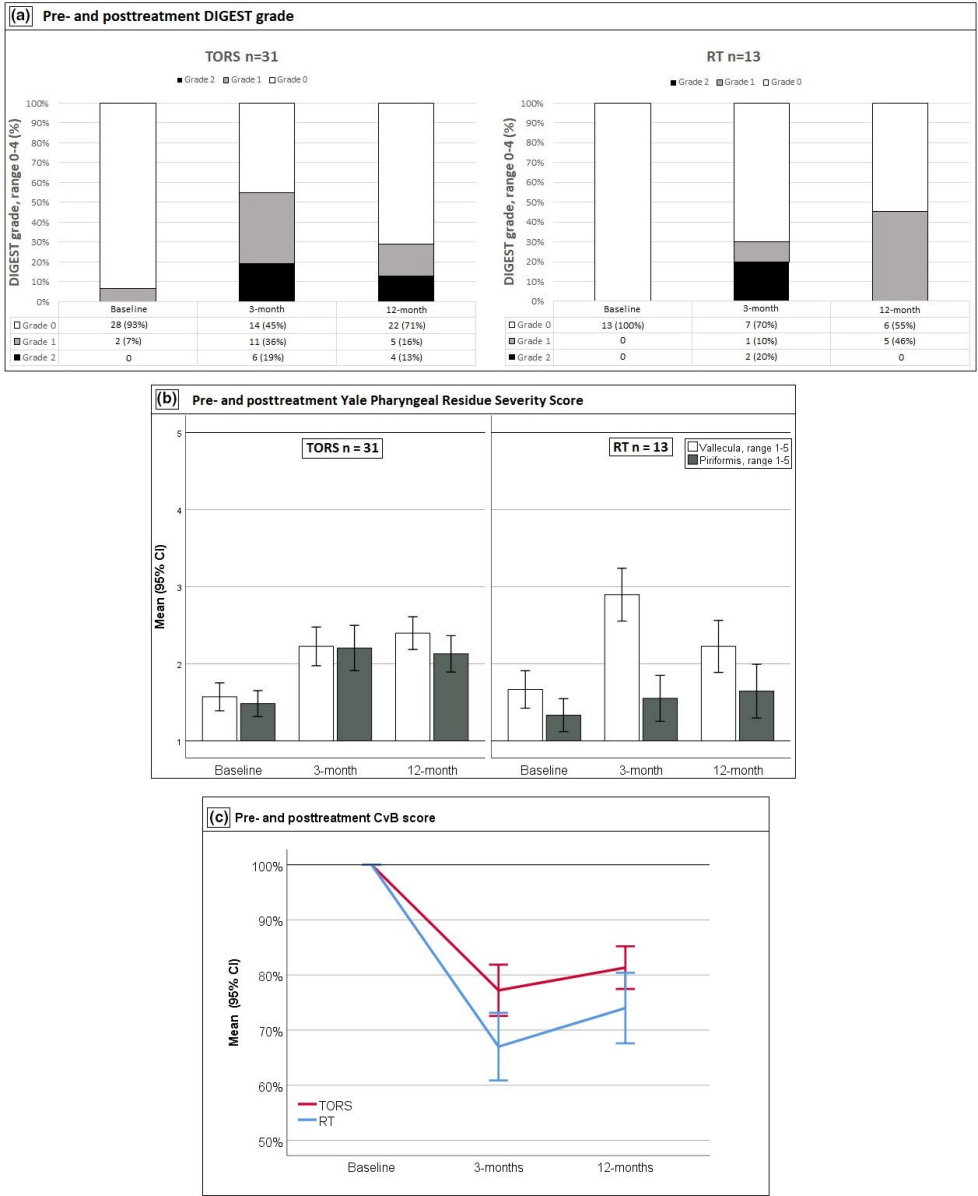
Pre‐ and posttreatment imaging studies results. A, Pre‐ and posttreatment DIGEST grade paneled by treatment. Grade 0 = no dysphagia, grade 1 = mild dysphagia, grade 2 = moderate dysphagia, grade 3 = severe dysphagia, and grade 4 = life threatening dysphagia. B, Pre‐ and posttreatment Yale Pharyngeal Residue Severity Score paneled by treatment. C, Pre‐ and posttreatment CvB score (%) paneled by treatment. CvB score is set at 100% at baseline at subsequent visits patients are asked to compare current function to their baseline (pre‐illness) function. The accessory nerve was sacrificed during neck dissection of one patient (3.2%) in the TORS group, who had notably worse results compared to the rest of the group. RT, Radiotherapy; TORS, Trans Oral Robotic Surgery

Comparison of baseline to 12‐month follow‐up scores showed significantly higher DIGEST scores (worse function) in both treatment groups, with the highest grade at any time point being 2 (moderate dysphagia). In the TORS group 26% of patients had increased scores at 12‐month follow‐up (*r* = 0.5, 95% CI 0.12–0.62, *p* = 0.009) in the RT group it was 39% (*r* = 0.6, 95% CI 0.09–0.74, *p* = 0.025). The change in DIGEST scores was driven mainly by an increase in efficiency scores. In the TORS group, the increase was significant with an effect size of 0.50 (95% CI 0.19–0.94, *p* = 0.007), while it was 0.50 (95% CI −0.01–1.01, *p* = 0.063) in the RT group. DIGEST grades at each time point are shown in Figure 2A.

Four patients (9%) had DIGEST scores of 2 at 12‐month follow‐up (see Figure 2A). All four had TORS for palatine tonsillar malignancies, two had received adjuvant CRT.

In the FEES‐related scores, there was no significant change in PAS when comparing baseline to 12‐month follow‐up in either treatment group, but a significant increase (more retention) in Yale scores at the vallecula (*r* = 0.5, 95% CI 0.55–1.10, *p* > 0.001) in the TORS and in the RT group (*r* = 0.4, 95% CI 0.17–1.10, *p* = 0.009). The TORS group also showed significant increase (more retention) at the piriform sinus (*r* = 0.5, 95% CI 0.39–0.89, *p* > 0.001) with no significant change in the RT group (*r* = 0.3, 95% CI −0.06–0.73, *p* = 0.108). Retention scores at each time point are shown in Figure 2B.

### 3.3 CvB scale

At baseline (pre‐illness), the CvB score was pre‐defined as 10 corresponding to 100% functionality. Scores for each treatment group at each time point is shown in Figure 2C.

Comparing baseline to 12‐month follow‐up scores showed significant declines (worse function) in both treatment groups with a mean decline of 19% in the TORS group (95% CI of change −25.7 to −11.7, *p* > 0.001) and 28% in the RT group (95% CI of change of −39.0 to −15.6, *p* > 0.001).

### 3.4 QoL

**FIGURE 3:**
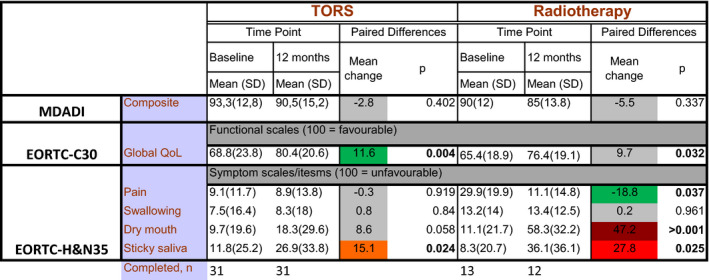
Heatmap of all subscales and composite scores that showed statistically significant or clinically meaningful changes from baseline to 12‐month follow‐up for MDADI, EORTC‐C30, and EORTC H&N35. EORTC‐C30, European Organization for Research and Treatment of Cancer Quality of Life Questionnaire Core Module; EORTC‐H&N35, EORTC Head & Neck Module; MDADI, MD Anderson Dysphagia Index; TORS, Trans Oral Robotic Surgery. X‐scale based on four questions added onto the questionnaire, when it was translated into Swedish (please see Appendix for the questions). *p* values under 0.005 are marked in bold. Color codes show changes bigger than the minimal clinically important difference (CID, set as 10% of the maximum instrument score if not otherwise stated in methods). Grey = no clinically important change, dark green = improvements between 11 and 20% more than CID, orange = decline between 11 and 20% more than CMI, red = decline between 21 and 30% more than CMI, dark red = decline between 41 and 50 more than CMI

Subscales and composite scores, corresponding to the functional outcomes (pain, swallowing function, and saliva flow rate) from baseline to 12‐month follow‐up for MDADI, EORTC‐C30, and EORTC H&N35 are shown in Figure 3. Full results are listed in Appendix.

### 3.5 MDADI

When comparing baseline to 12‐month follow‐up scores, in a post‐hoc analysis, we found only four question scores showed significant changes. In the TORS group, it was only two question: P7 (*It takes me longer to eat because of my swallowing problem*; mean change 0.7, 95% CI 0.07–1.35, *p* = 0.031) and the Swedish translation added item X4 (*I have to use liquids to clear when I eat*; mean change 0.7, 95% CI 0.08–1.23, *p* = 0.029). In the RT group, it was: P4 *(I feel that I am swallowing a huge amount of food*, mean change 1.0, 95% CI −0.01–2,01, *p* = 0.048), P7 (see above, mean change 1.25, 95% CI 0.01–2.50, *p* = 0.040), X2 (*the food sticks when I swallow*, mean change 1.2, 95% CI 0.36–1.97, *p* = 0.023), and X3 (*swallowing is difficult because I have a dry mouth*, mean change 1.8, 95% CI 0.94–2.73, *p* = 0.008).

### 3.6 Shoulder function

**FIGURE 4:**
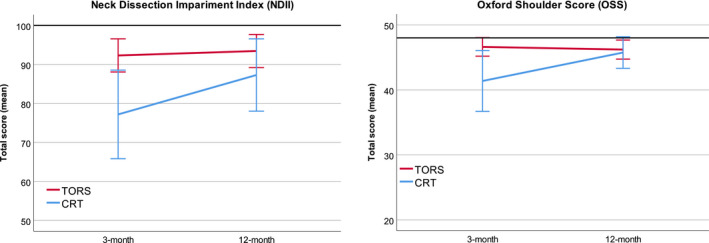
Neck Dissection Impairment Index and Oxford Shoulder Score total scores. Bars, 95% confidence interval; black line, maximum (best) possible score; TORS, Trans Oral Robotic Surgery; RT, radiotherapy

Total scores for both questionnaires panel by treatment were similar and mostly unchanged (Figure 4).

## 4 DISCUSSION

QoL and functional outcomes should be a factor in OPSCC treatment decisions, with emphasis on individualized treatment as patients will have different functional requirements affecting their QoL. In this descriptive, hypothesis‐generating feasibility study we present a comprehensive examination of patients treated with either TORS or RT focused on known adverse effects and complications of both modalities.

Xerostomia is a well‐known adverse effect of RT and in a comparative study Xu et al^19^ reported significantly worse xerostomia scores for patient treated with RT (as single or part of multi‐modality treatment) compared to surgery alone. For patients treated with RT, we found declined saliva flow rates and reported xerostomia scores at the 12‐month follow‐up. For patients treated with TORS, we found, a slight but significant increase (improvement) in stimulated salivary flow rate and an increase in the sticky saliva subscale of the EORTC‐H&N35 at the 12‐month follow‐up. Taken together, this may represent a change in the viscosity of the saliva due to a decreased clearing in the resected area but warrant further studies.

Longitudinal data on imaging based swallowing assessments after TORS is still rare. Lazarus et al^20^ only reported none to mild dysphagia (DIGEST <2) on MBS evaluations comparing pre‐ to 1‐month postoperative scores, while Hutcheson et al^21^ found a prevalence of moderate to severe dysphagia (DIGEST ≥2) of 6.7% for patients treated with TORS and 15.9% for patients treated with RT when assessing 257 patients treated for oropharyngeal cancer 3 to 6 months after treatment. We found, the cohort to generally have good swallowing function, on imaging studies, with no severe dysphagia within 1 year and a low prevalence of moderate dysphagia. However, both treatment groups showed significant worsening on imaging based swallowing assessments at 12‐month follow‐up. This is corroborated by the significant declines (worse function) in patients’ perception of their swallowing recovery per CvB scores in both treatment groups as well as image‐based assessments showing a significant increase in pharyngeal retention.

Currently, there is a drive to examine OPSCC treatment modalities with QoL endpoints (e.g. ORATOR,^22^ QoLATI^23^ and PATHOS^24^). The most commonly used QoL questionnaires including the ones used in this study (MDADI, EORTC‐C30, and ‐H&N35) were developed on a patient population predominantly treated with RT or CRT. These measures may, therefore, not fully represent all issues relating to surgical resection. In all three QoL questionnaires, in this study, the only change for the worse, we found in the TORS group, was in the “sticky saliva” subscale. Furthermore, we found improvements in the EORTC‐C30 “global QoL,” which were both significant and clinically important. When examining individual questions of MDADI, we only found a statistically significant difference in scores for two question (“It takes me longer to eat because of my swallowing problem” and “I have to use liquids to clear when I eat”). While this likely reflects good QoL after TORS, there is a possibility that more surgically oriented questionnaires might uncover other factors that impact QoL that are simply not detected in QoL instruments largely validated in non‐surgically treated cohorts. In contrast the RT group, had significant and clinically important worsening in “X‐scale” of MDADI (mean difference 16.3), as well as before mentioned “dry mouth” and “sticky saliva” of the EORTC‐H&N35, with clinically important but not significant worsening in “senses problems” (mean difference 12.5).

Treatment related shoulder impairment is a potential adverse effect of both surgical and RT treatment of the neck,^19^ we included questionnaires to explore the scale of impairment. We used the NDII and the OSS and found both questionnaires to reflect some impairment at 3‐month follow‐up in the RT group which improved at 12‐month follow‐up. The shoulder questionnaires were not part of the baseline assessment so we cannot conclude if baseline function was recovered but the TORS group showed very stable, low levels of impairment. The difference in impairment might be explained by the higher percentage of RT patients who received bilateral neck *radiation* compared to TORS patients who underwent bilateral neck *dissections*, 69% versus 45%, respectively.

In a post hoc analysis, the five TORS patients who received CRT within a year of treatment (four as adjuvant and one as salvage treatment) were found to have the worst outcomes compared to single modality TORS and CRT with regards to swallowing function (imaging assessed and CvB score) as well as QoL scores with similar shoulder function results to the CRT group.

This study had several limitations which should caution the interpretation of the results. The small sample size, particularly in the RT group, limits power to detect significant changes in outcome measures. Selection bias because only T1‐2 and N0‐1 were considered for TORS. Lastly, while all but one eligible TORS patients were included in this study, patients receiving RT were less inclined to participate evident in the low number of patients enrolled compared to eligible patients in this group possibly skewing the results.

The most notable strength of this study is the comprehensive examination of this OPSCC cohort, collecting multiple measures at set time points. These outcomes will help to inform a larger scale, preferably randomized study to more accurately assess treatment related differences and severity in the adverse effects. We found several significant differences between baseline and 12‐months follow‐up scores with overall good functional and QoL outcomes 1 year after treatment for OPSCC regardless of modality.

## CONFLICTS OF INTEREST

The authors declare no conflicts of interest.

## AUTHOR CONTRIBUTIONS

Susanne Scott: substantial contributor to the conception, design of the work, acquisition, analysis and interpretation of data for the work, as well as drafting the work, approving final version, and agree to full accountability. Anne Kathrine Ø. Madsen: substantial contributor to the conception, design of the work, acquisition of data for the work, critical revision, approving final version, and agree to full accountability. Niclas Rubek: substantial contributor to the conception, design of the work, acquisition of data for the work, critical revision, approving final version, and agree to full accountability. Birgitte W. Charabi: substantial contributor to the conception, design of the work, critical revision, approving final version, and agree to full accountability. Irene Wessel: substantial contributor to the conception, design of the work, critical revision, approving final version, and agree to full accountability. Sara Fredslund Hadjú: substantial contributor to the acquisition of data for the work, critical revision, approving final version, and agree to full accountability. Claus V. Jensen: substantial contributor to the acquisition of data for the work, critical revision, approving final version, and agree to full accountability. Sarah Stephen: substantial contributor to the interpretation of data for the work, critical revision, approving final version, and agree to full accountability. Joanne M. Patterson: substantial contributor to the interpretation of data for the work, critical revision, approving final version, and agree to full accountability. Jeppe Friborg (JF): substantial contributor to the conception, design of the work, critical revision, approving final version, and agree to full accountability. Kathrine A. Hutcheson: substantial contributor to the conception, design of the work, analysis and interpretation of data for the work, as well as revising the work critically, and approving final version. Agree to full accountability. Henrik Kehlet: substantial contributor to the conception, design of the work, interpretation of data for the work, as well as revising the work critically, and approving final version. Agree to full accountability. Christian von Buchwald: substantial contributor to the conception, design of the work, interpretation of data for the work, as well as revising the work critically, and approving final version. Agree to full accountability.

## TRIAL REGISTRATION AND ETHICAL APPROVAL

The study was conducted in accordance with the Declaration of Helsinki and was approved by the local ethical committee (H‐1‐2014‐033 and H‐17015164). Written informed consent was obtained. The study protocol has been published on clinicalTrials.gov (NCT03418909). Long‐term Quality of Life & Functional Outcomes After Treatment of Oropharyngeal Cancer.

## Data Availability

The data that support the findings of this study are available from the corresponding author upon reasonable request.

## SALIVARY FLOW RATE ‐ METHODS

Salivary flow rate was assessed using the drooling method with 5 min of unstimulated collection time followed by 1 min of stimulation by chewing a paraffin gum after this was discarded a stimulated sample was collected over a 5‐min period. Samples were collected between 12 am and 1 pm. Prior to sampling the patient had been fasting for an hour.

Saliva sample size was estimated under the assumption that 1 milliliter (ml) of saliva weighs one gram (g).^23^

## SWALLOWING FUNCTION ‐ METHODS

VF was performed using standard fluoroscopy equipment with the patient sitting during the study. Visipaque and barium paste were used as contrast agents. Liquid boluses were administered on a syringe. Each bolus was analyzed using DIGEST and Penetration Aspiration Scale (PAS) was assessed throughout the recorded swallows for each bolus. DIGEST ranges from grade 0 (best function) to 4 (worst). It was developed specifically for head and neck cancer patients.^9^

FEES was performed using a fiber‐endoscope connected to a recording device (MediCap® USB200 or 300) recording both video and audio. Only an anecdotal number of examinations were performed with the use of local anesthetic agents (Lidocaine). All liquid boluses were administered on a syringe. Each bolus was analyzed with regards to the Yale Pharyngeal Residue Severity Rating Scale (Yale) score at the vallecula and the piriform sinus. Assessments were made when the patient felt they had swallowed the bolus regardless of how many swallows were used to get there. PAS was assessed throughout all attempts to swallow the bolus.

If a bolus could not be swallowed maximum points were awarded for retention/residual and PAS left as a missing value, in both VF and FEES.

MDADI *x*‐scale questions.

*X*‐scale questions (translated into English from Danish):
X1: It hurts when I eat/drink/swallow.
X2: The food sticks when I swallow.
X3: Swallowing is difficult because I have a dry mouth.
X4: I must use liquids to clear when I eat.

**FIGURE A1.** Baseline and 12‐month follow‐up results for MDADI, EORTC‐C30, and ‐H&N35 for each treatment group. EORTC‐C30, European Organization for Research and Treatment of Cancer Quality of Life Questionnaire Core Module; EORTC‐H&N35, EORTC Head & Neck Module; MDADI, MD Anderson Dysphagia Index; TORS, Trans Oral Robotic Surgery. *X*‐scale based on four questions added onto the questionnaire, when it was translated into Swedish (please see below for the questions). *p* values under 0.005 are marked in bold. Color codes show changes bigger than the minimal clinically important difference (CID, set as 10% of the maximum instrument score if not otherwise stated in methods). Grey = no clinically important change, dark green = improvements between 11% and 20% more than CID, orange = decline between 11 and 20% more than CMI, red = decline between 21% and 30% more than CMI, dark red = decline between 41 and 50 more than CMI

**FIGURE A2.** Longitudinal MDADI results at all three timepoints paneled by treatment
